# Identification of amino acid residues involved in the dRP-lyase activity of human Pol ι

**DOI:** 10.1038/s41598-017-10668-5

**Published:** 2017-08-31

**Authors:** Nataliya Miropolskaya, Ivan Petushkov, Andrey Kulbachinskiy, Alena V. Makarova

**Affiliations:** Institute of Molecular Genetics, Russian Academy of Sciences, Kurchatov sq. 2, 123182 Moscow, Russia

## Abstract

Besides X-family DNA polymerases (first of all, Pol β) several other human DNA polymerases from Y- and A- families were shown to possess the dRP-lyase activity and could serve as backup polymerases in base excision repair (Pol ι, Rev1, Pol γ and Pol θ). However the exact position of the active sites and the amino acid residues involved in the dRP-lyase activity in Y- and A- family DNA polymerases are not known. Here we carried out functional analysis of fifteen amino acid residues possibly involved in the dRP-lyase activity of human Pol ι. We show that substitutions of residues Q59, K60 and K207 impair the dRP-lyase activity of Pol ι while residues in the HhH motif of the thumb domain are dispensable for this activity. While both K60G and K207A substitutions decrease Schiff-base intermediate formation during dRP group cleavage, the latter substitution also strongly affects the DNA polymerase activity of Pol ι, suggesting that it may impair DNA binding. These data are consistent with an important role of the N-terminal region in the dRP-lyase activity of Pol ι, with possible involvement of residues from the finger domain in the dRP group cleavage.

## Introduction

Human DNA polymerase iota (Pol ι) belongs to the Y-family of translesion DNA polymerases and demonstrates very low accuracy of DNA synthesis. The high error rate of Pol ι is a result of the special organization of the active site^1, 2^ which is adapted to bypass a variety of DNA lesions^3^, including bulky carcinogenic lesions^4–9^ and interstrand DNA cross-links^10^. The DNA polymerase activity of Pol ι is stimulated by Mn^2+^ ions^11, 12^. In addition to the DNA polymerase activity, human Pol ι has an intrinsic 5′-deoxyribo﻿se-5-phosphate lyase activity (dRP-lyase activity)^13, 14^. Pol ι carries out efficient DNA synthesis on gapped DNA substrates and in reactions reconstituted with uracil-DNA glycosylase, AP-endonuclease and ligase can repair DNA^13, 15^. Moreover, Pol ι is able to complement *in vitro* the short-patch base excision repair (BER) deficiency of Pol β null cell extracts^16^. Extracts from cells with downregulated Pol ι also show reduced BER activity^14^. In addition, Pol ι binds to chromatin under oxidative stress and interacts with the BER factor XRCC1^14^. Altogether these data support a role of Pol ι in certain types of BER.

**Figure 1:**
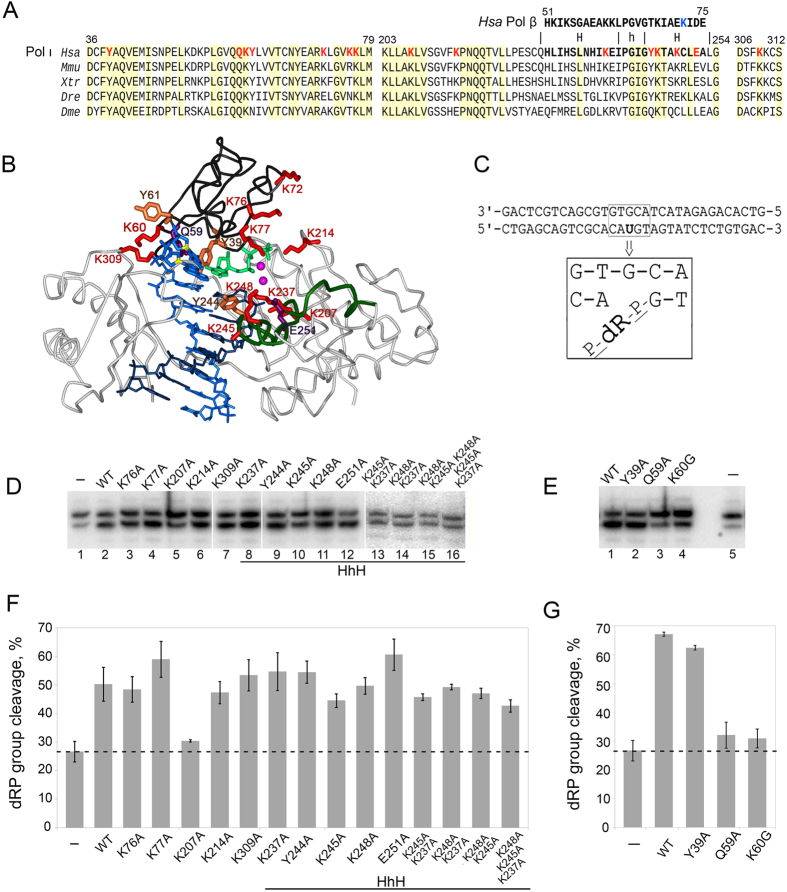
Effects of amino acid substitutions on the dRP-lyase activity of Pol ι. (**A**) Alignments of the fingers and thumb domains of Pol ι from various eukaryotes. *Hsa*, *Homo sapiens*; *Mmu*, *Mus musculus*; *Xtr*, *Xenopus tropicalis*; *Dre*, *Danio rerio*; *Dme*, *Drosophila melanogaster*. Amino acid numbering corresponds to human Pol ι. Pol ι residues analyzed in this study are red-colored. The sequence of the HhH motif of the N-terminal lyase domain in Pol β is shown above the HhH motif of the thumb domain in Pol ι. The catalytic residue K72 involved in the dRP-lyase activity in human Pol β is shown in light blue color. (**B**) Structure of Pol ι in complex with DNA template (with 1,N^6^-ethenoadenine in the active site; light blue), DNA primer (dark blue) and incoming dNTP (light green) (2DPI^6^). The fingers domain is dark gray; the HhH motif in the thumb domain is green. Pol ι lysine residues analyzed in this study are red-colored; Y39, Y61 and Y244 are shown in orange; Q59 and E251 are purple. (**C**) The structure of the oligonucleotide substrate with the 5′-dRP group used in this study. (**D**) and (**E**) Gel images of the dRP-lyase activity of wild-type and mutant Pol ι variants. Lanes 1 in D and 5 in E (“−”) show spontaneous cleavage of the 5′-dRP group observed in the absence of Pol ι protein. The full-length gels are presented in Supplementary Figs 2 and 3. (**F**) and (**G**) Diagrams showing percentages of the 5′-dRP group cleavage by Pol ι variants. The dashed line shows the level of spontaneous cleavage observed in the absence of Pol ι. Substitutions K76A, K77A, K207A, K214A, K237A, Y244A, K245A, K248A, E251A, K309A, KK237/245AA, KK237/248AA, KK245/248AA, KKK237/245/248AAA were obtained in the catalytic core of Pol ι consisting of residues 1–420 (**D** and **F**). Substitutions Y39A, Q59A, K60G, Y61A and K72A were obtained in the full-length Pol ι protein (**E**, **G** and Supplementary Fig. 1).

Removal of a 5′-dRP group by Pol ι likely proceeds via β-elimination mechanism^13^ involving formation of a Schiff base intermediate between an enzyme primary amine (usually originating from a lysine residue) and the Cl′ atom of the deoxyribose phosphate^17–19^. Nevertheless, the mechanism of dRP-lyase activity of Pol ι is not well understood. In particular, the site of the dRP-lyase activity and amino acid residues involved in catalysis are yet to be determined. Based on sequence alignments with Pol β, it was suggested that the site of the dRP-lyase activity is located in a helix-hairpin-helix motif (HhH-motif) of the thumb domain of Pol ι (Fig. 1A and B)^13, 14^. However the role of this motif in Pol ι was not biochemically established.

In this work we tested the effects of fifteen amino acid substitutions, including all lysine residues located around the DNA-binding cleft of Pol ι, on its dRP-lyase activity. Tested mutations were located in the HhH motif of the thumb domain (K237A, Y244A, K245A, K248A, E251A) and in the fingers domain around the DNA polymerase active site (Y39A, Q59A, K60G, Y61A, K72A, K76A, K77A). Additionally, we mutated lysine residues potentially interacting with DNA and selected based on available 3D structures of Pol ι-DNA complexes (K207A, K214A, K309A) (Fig. 1A and B). We analyzed the effects of these mutations on Pol ι catalysis and for the first time identified amino acid residues critical for the removal of 5′-dRP group.

## Results

### Residues Q59, K60 and K207 are required for the dRP-lyase activity of Pol ι

Human Pol ι is a 80 kDa protein consisting of 715 amino acid residues. First 420 amino acid residues are sufficient for the DNA polymerase activity of Pol ι and referred to as the catalytic core^1, 20^. We found that the catalytic core also retains the dRP-lyase activity, which was slightly reduced compared to the wild-type Pol ι (compare Fig. 1D, lane 2 and Fig. 1E, lane 1). These data suggest that the dRP-lyase active site is located in the N-terminal half of Pol ι protein.

We next analyzed the effects of fifteen amino acid substitutions on the dRP-lyase activity of Pol ι. The choice of residues was based on the predictions made using sequence alignment of Pol ι and Pol β and previous cross-linking experiments^13, 16^, as well as available structural data^1, 2, 6^. The positions of all residues selected for mutagenesis are illustrated in Fig. 1A and B. To facilitate protein purification we used the catalytic core of Pol ι for the majority of amino acid substitutions (see Materials and Methods and Fig. 1 legend for details).

In Pol β the HhH motif of the N-terminal 8 kDa lyase domain catalyzes the excision of the 5′-dRP group from DNA^19, 21, 22^. Y-family DNA polymerases lack the N-terminal lyase domain. However the thumb domain of Pol ι contains a HhH motif consisting of residues 228–252 and showing similarity with the HhH motif of the N-terminal lyase domain of Pol β based on sequence alignment^13, 14^ (Fig. 1A). To probe the function of this HhH motif in the dRP-lyase activity of Pol ι we tested the ability of Pol ι variants with amino acid substitutions K237A, Y244A, K245A, K248A and E251A to cleave the 5′-dRP group. In addition, we analyzed double and triple substitutions of these residues (KK237/245AA, KK237/248AA, KK245/248AA, KKK237/245/248AAA).

Surprisingly, we found that analyzed residues in the HhH motif are dispensable for the dRP-lyase activity by Pol ι. Substitutions K237A, Y244A and E251A did not affect the dRP-lyase activity (Fig. 1D, lanes 8, 9 and 12, and Fig. 1F). Nearly wild-type activity (80–90%) was observed for the K245A and K248A Pol ι variants (Fig. 1D, lanes 10 and 11, and Fig. 1F). The double and triple amino acid substitutions of lysine residues also did not abrogate the dRP-lyase activity of Pol ι (Fig. 1D, lanes 13–16, and Fig. 1F).

It was shown previously that the N-terminal region comprising the first 78 residues is required for the Schiff base formation of Pol ι^16^. We therefore tested the role of residues Y39, Q59, K60 and Y61 (conserved in vertebrates, Fig. 1A) in this region in the dRP-lyase activity. These residues are located in the DNA polymerase active site of Pol ι and contact the DNA template and incoming nucleotides during catalysis^1, 2, 6^. Substitutions Y39A, Q59A, K60G and Y61A were obtained previously in the full-length Pol ι protein; substitutions Y39A and Q59A were shown to significantly affect the DNA polymerase activity of Pol ι^12^. We found that substitutions Q59A and K60G dramatically reduced the dRP-lyase activity by Pol ι, almost to the level of spontaneous cleavage (Fig. 1E, lanes 3 and 4, and Fig. 1G). Substitution Y39A did not significantly affect the dRP-lyase activity of Pol ι (Fig. 1E, lane 2 and Fig. 1G), while substitution Y61A decreased the activity (Supplementary Fig. 1). Other three lysine residues K72, K76 and K77 located in this region were shown to be dispensable for the dRP-lyase activity (Fig. 1D,F and Supplementary Fig. 1).

Based on available structural data we also chose for analysis three lysine residues that contact DNA and could potentially be involved in the dRP-lyase activity as catalytic nucleophiles: K207, K214, and K309 (Fig. 1A and B). While substitutions K214A and K309A did not affect the dRP-lyase activity of Pol ι (Fig. 1D, lanes 6, 7, and Fig. 1F), substitution K207A strongly decreased the 5′-dRP group cleavage (Fig. 1D, lane 5, and Fig. 1F).

### Effects of K60G, K207A and K245A substitutions on the Schiff base intermediate formation

We next compared the ability of mutant variants of Pol ι to form the Schiff base intermediate during β-elimination, which can be covalently trapped by reduction with NaBH_4_. We compared the amounts of NaBH_4_-stabilized cross-linked products formed with the 5′-dRP group by the wild-type and mutant Pol ι variants.

**Figure 2:**
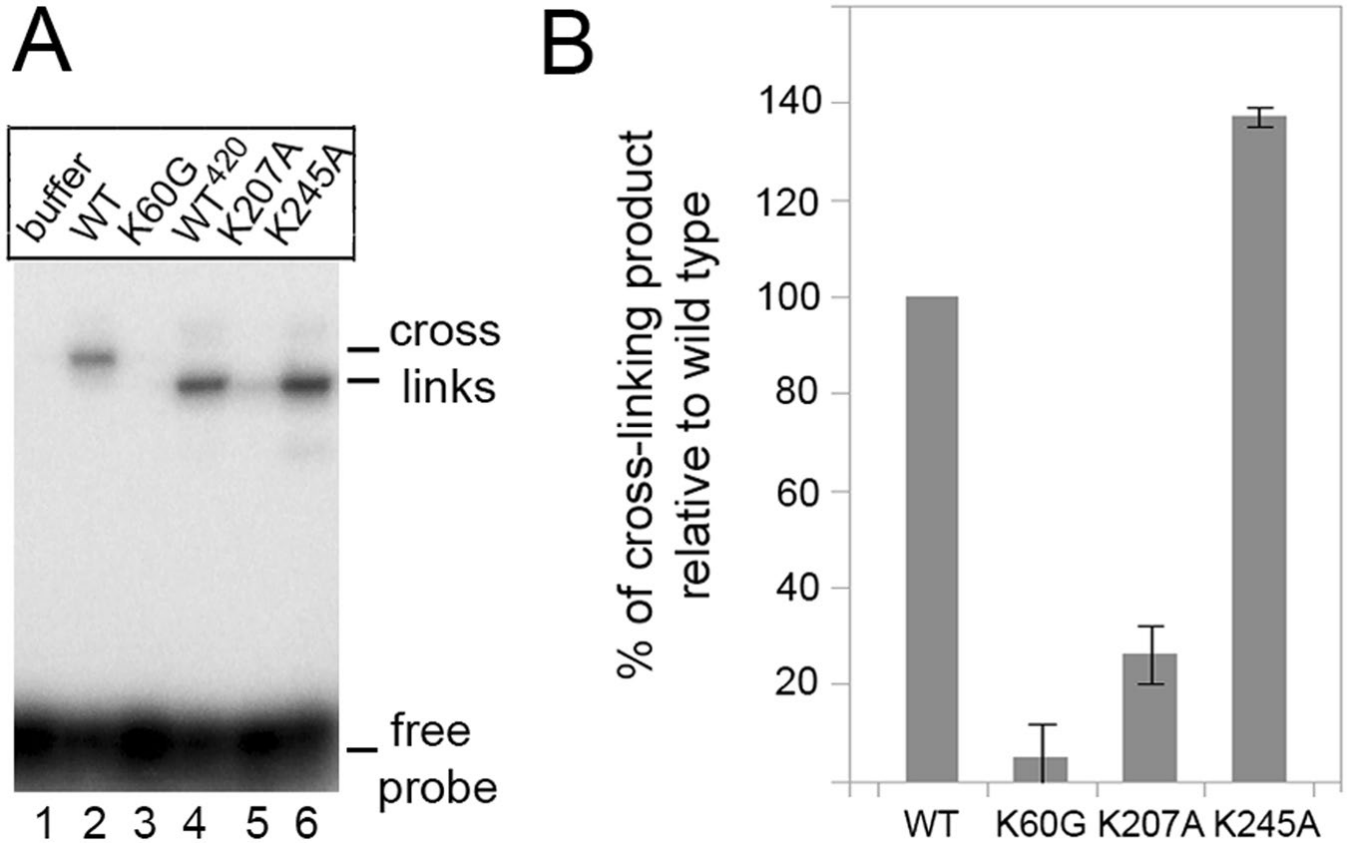
Effect of Pol ι amino acid substitutions on the Schiff base intermediate formation. (**A**) Gel image of cross-linked products formed by wild-type and mutant Pol ι variants after NaBH4-treatment. The truncated variant of wild-type Pol ι consisting of the first 420 amino acid residues is designated as WT^420^. (**B**) The diagram of the calculated percentages of cross-linked products formed by Pol ι variants (relative to the wild-type enzyme).

It was shown that a control K245A substitution in the HhH motif, which did not affect the dRP-lyase activity (see above), also did not impair the Schiff base formation by Pol ι (Fig. 2A, compare lanes 4 and 6, and Fig. 2B). In contrast, the K60G and K207A substitutions significantly decreased the amounts of cross-linked products (Fig. 2A, lanes 3 and 5, and Fig. 2B). The strongest decrease in the Schiff base formation was observed for the K60G Pol ι mutant.

### Effects of K60G and K207A substitutions on the DNA polymerase activity of Pol ι

**Figure 3:**
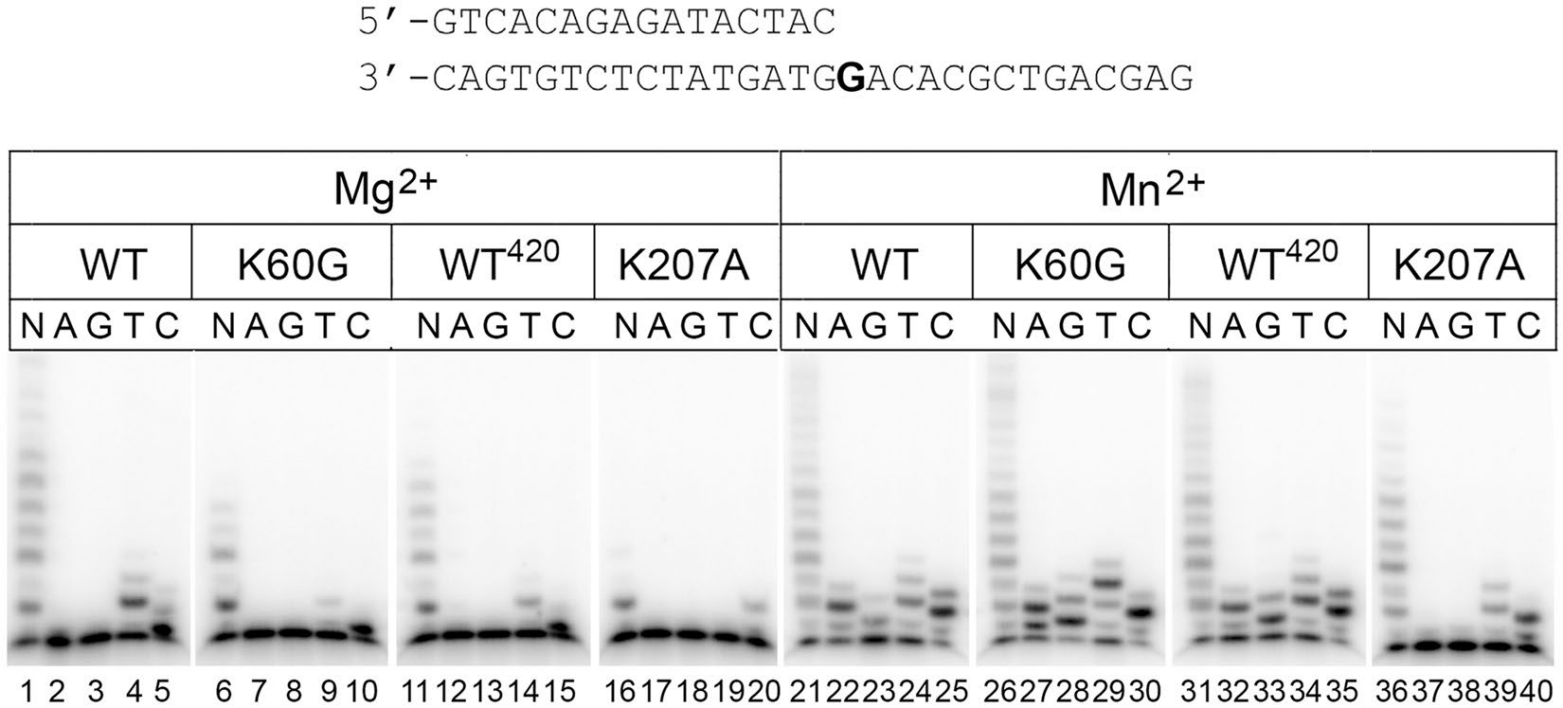
Effects of amino acid substitutions on the DNA polymerase activity of Pol ι. The structure of the oligonucleotide substrate used to test the DNA polymerase activity is shown on top. Gel image of DNA polymerase activity of wild-type Pol ι and its mutant variants is shown below. Primer extension reactions were performed in the presence of Mg^2+^ and Mn^2+^ ions. All four dNTPs (N) or individual dNTP (A, G, T, C) were added to the reaction. The full-length and truncated (WT^420^) variants of wild-type Pol ι were used for comparisons with the K60G and K207A mutants, respectively.

According to the Pol ι catalytic core structure, residues K60 and K207 make direct contacts with DNA^1, 2, 6^ (Fig. 1B). Therefore the loss of the dRP-lyase activity by the K60G and K207A Pol ι mutants could be caused by the loss of protein-DNA contacts. To reveal whether these substitutions generally impair DNA binding, we measured the DNA polymerase activity of these mutants in primer extension reactions on an oligonucleotide substrate (Fig. 3). Since DNA synthesis by Pol ι is stimulated by manganese ions, the reactions were performed in the presence of either Mg^2+^ or Mn^2+^ ions. In Mg^2+^ reactions, the wild-type Pol ι incorporated opposite template G not only dCTP but also dTTP. These agrees with previous reports on the low fidelity of DNA polymerase synthesis by Pol ι; dTTP misincorporation may be also due to transient template misalignment and positioning of the next template A into the active site. In agreement with published data, Mn^2+^ stimulated Pol ι activity. The activity of the K60G variant was reduced only in the presence of Mg^2+^ (Fig. 3, lanes 6–10) and was similar to the wild-type Pol ι in the presence of Mn^2+^ ions (Fig. 3, lanes 26–30). In contrast, the K207A variant was defective in DNA synthesis with both Me^2+^ cofactors (Fig. 3, lanes 16–20 and 36–40). Moreover, the K207A substitution also changed the spectrum of dNTP incorporation by Pol ι. In particular, it abrogated the incorporation of dATP and dGTP opposite template G and thus increased the fidelity of DNA synthesis by Pol ι (Fig. 3, lanes 37, 38). Thus, substitution K207A shows a stronger effect on the DNA polymerase activity compared to the K60G substitution.

## Discussion

In X-family DNA polymerases Pol β and Pol λ the dRP-lyase active site is located in the HhH motif of the N-terminal 8 kDa lyase domain^19, 21–24^. Human A-family Pol γ and Pol θ and Y-family Pol ι and Rev1 polymerases also possess the dRP-lyase activity^13, 25–27^. These polymerases lack a significant amino acid sequence similarity with Pol β in the N-terminal domains and amino acid residues involved in the dRP-lyase activity are not determined. Here, we for the first time analyzed the functional role of candidate amino acid residues in the dRP-lyase activity of Pol ι and identified residues required for the excision of the 5′-dRP group.

It was previously proposed that the active site of the dRP-lyase in Pol ι may be located in the thumb HhH motif that comprises residues 228–252 (Fig. 1A,B). In particular, it was speculated that Pol ι residue K237 may serve as the orthologue of Pol β residue K72 during the β-elimination reaction^14^. However, we showed that neither of tested substitutions in the HhH motif (including substitutions of lysine residues K237A, K245A and K248A) significantly affected the dRP-lyase activity or Schiff base formation (as tested for the K245A substitution). Moreover, double and triple substitutions of residues K237, K245 and K248 also did not abrogate the dRP-lyase activity of Pol ι. Our observations are in agreement with data of Dr. Roger Woodgate laboratory, who have also recently shown that single K237A, K245A and K248A Pol ι mutants do not compromise the dRP-lyase activity of Pol ι (personal communication) Therefore, it is likely that the HhH motif of the thumb domain does not play an important role in the 5′-dRP group cleavage in human Pol ι.

Previous experiments with controlled proteolysis mapped the cross-link formed after formation of the Schiff base with the 5′-dRP group in a protease-resistant 40 kDa domain which comprises residues 79–445 of Pol ι^16^. However, this 40 kDa domain alone was not capable of the dRP lyase activity. The N-terminal 8 kDa region containing residues 1–78 was shown to be necessary for the Schiff base formation with the 40 kDa domain^16^.

Consistent with this observation, we showed that mutations of conservative residues Q59 and K60 in the N-terminal region lead to the loss of the dRP-lyase activity in Pol ι. Substitution of residue Y61 also decreases this activity, probably by affecting the conformation of the adjacent K60 residue. Recently we showed that mouse Pol ι missing the region encoded by exon 2 (corresponds to residues 14–55 in human Pol ι) also lacks the dRP-lyase activity^28^. It is likely that deletion of this region impairs the correct folding of the N-terminus of Pol ι required for this activity. These data emphasize the essential role of the N-terminal region in the dRP-lyase activity of Pol ι.

Residues Q59 and K60 are located in the DNA polymerase site in the fingers domain of Pol ι. In addition, substitution of residue K207 from the palm domain was shown to affect the dRP-lyase activity. Substitutions of both lysines K60 and K207 dramatically decrease the cross-link formation with the 5′-dRP group and to various extent diminish the DNA polymerase activity of Pol ι. At present, we cannot draw a clear conclusion on whether any of these lysines play a catalytic role in the 5′-dRP group cleavage. However, the K60 residue is more suitable for this role. Despite the fact that K60 is located within the DNA polymerase active site, substitution of this residue had a smaller effect on the DNA polymerase activity of Pol ι and stronger effect on the Schiff base formation than substitution of residue K207. Thus, the observed changes in the K207A Pol ι activity may be attributed to significant defects in DNA binding while residue K60 might be directly participating in the reaction.

It should be noted that participation of the K60 residue in the 5′-dRP group cleavage would require significant reorientation of the DNA substrate in comparison with available structures (see Fig. 1B; the C1′ atoms in the DNA 5′-end closest to K60 are shown in yellow). This reorientation requirement may explain why the dRP-lyase activity of Pol ι is ~30-fold weaker compared to Pol β^16^. The hypothesis that the dRP-lyase active site is located in the N-terminal 8 kDa region also contradicts previous studies, which localized the site of the dRP-group cross-linking in the 40 kDa domain spanning residues 79–445^16^. We speculate that a nonspecific cross-link between the 40 kDa domain and the 5′-dRP group might interfere with precise mapping of specific contacts, while detection of the 8 kDa region cross-linking might be difficult because its position coincided with the intense signal of free DNA probe. Alternatively, residues Q59 and K60 might indirectly affect the dRP-lyase reaction, by stabilizing the correct DNA orientation or protein conformation required for the dRP-group cleavage. Future structural studies are required to verify the exact role of the N-terminal region in the dRP-lyase activity of Pol ι.

## Methods

### Purification of human Pol ι and its mutant variants

The production and purification of Pol ι proteins from *S. cerevisiae* protease-deficient strain BJ 2168 was carried out as described^28^. Proteins were eluted and stored at −80 °C in elution buffer containing 30 mM HEPES pH 8.0, 8% glycerol, 100 mM KCl, 5 mM K_2_HPO_4_/KH_2_PO_4_ pH 8.0, 1 mM DTT, 30 mM glutathione. Full-length wild-type Pol ι, full-length Pol ι variants with amino acid substitutions Y39A, Q59A, K60G and Y61A as well as truncated Pol ι encoding for the first 420 amino acids were obtained earlier^12, 20^. Full-length Pol ι variant with amino acid substitution K72A was obtained in this work using site-directed mutagenesis. Pol ι variants with single, double and triple amino acid substitutions K76A, K77A, K207A, K214A, K237A, Y244A, K245A, K248A, E251A, K309A, KK237/245AA, KK237/248AA, KK245/248AA, KKK237/245/248AAA were obtained in the truncated Pol ι variant by site-directed mutagenesis and purified in the same way. All proteins were more than 95% pure.

### DNA oligonucleotide substrates

DNA oligonucleotides were synthesized by Syntol and DNA Synthesis (Moscow, Russia) and were PAGE-purified prior to use. To prepare radiolabeled oligonucleotide duplex DNA substrate for testing dRP-lyase activity (Fig. 1C), 34-mer oligonucleotide containing uracil at position 16 was 3′-labeled by terminal deoxynucleotidyl transferase using [^α−32^P]dATP for 3 min at 37 °C. This strand was then annealed to complementary 34-mer oligonucleotide by heating at 90 °C for 3 min and slow cooling to RT. Then uracil-containing DNA substrate was treated with uracil-DNA-glycosylase (UDG) and AP-endonuclease (APE1) to create a nick with the 5′-dRP group as described^16^.

To obtain DNA substrate for DNA polymerase reactions 16-mer primer was 5′-labeled with [^γ−32^P]-ATP using T4 polynucleotide kinase and annealed to unlabeled 30-mer template oligonucleotide^12^.

### Detection of dRP-lyase activity

Testing of dRP-lyase activity was performed as described^16, 28^ with modifications. The reaction mixture contained 30 mM HEPES pH 7.5, 20 mM KCl, 5 mM MgCl_2_, 8% glycerol, 1 mM DTT, 100 nM DNA substrate, 250 nM full-length Pol ι proteins or 500 nM truncated Pol ι variants. After incubation at 37 °C for 30 min the reaction tubes were placed on ice, NaBH_4_ was added to a final concentration of 340 mM to stabilize DNA products and incubation was continued for 30 min on ice. The DNA products were ethanol precipitated, separated by 23% PAGE with 8 M urea in 1xTBE and visualized with Typhoon FLA 9500 scanner (GE Healthcare).

### Cross-linking of DNA to Pol ι

To prepare the covalently cross-linked Pol ι-DNA complex, the NaBH_4_ trapping technique was used^16, 18^. Briefly, the reaction mixture contained 50 mM Hepes pH 7.5, 20 mM KCl, 1 mM DTT, 5 mM MgCl_2_, 200 nM [^32^P]-labeled DNA substrate and 500 nM full-length Pol ι proteins or 1000 nM truncated Pol ι variants. After incubation at 22 °C for 5 min, NaBH_4_ was added to the reactions to 1 mM final concentration and reactions were incubated for 30 min on ice. Then equal volume of sample buffer (0.25 M Tris-HCl pH 6.8, 10% glycerol, 2% SDS, 2 mM 2-mercaptoethanol) was added and covalent complexes were resolved by SDS-PAGE on 4–20% Mini-PROTEAN TGX Precast Protein Gels (Bio-Rad). The Pol ι-DNA complexes were visualized with Typhoon FLA 9500 scanner.

### Primer extension DNA polymerase reactions

Standard primer extension reaction was carried out in 20 µl of reaction buffer containing 30 mM HEPES pH 7.8, 50 mM NaCl, 8% glycerol, 0.1 mg/ml bovine serum albumin, 40 nM DNA substrate, 100 μM each four or individual dNTPs (GE Healthcare). The concentrations of divalent metal salts were 0.15 mM for MnCl_2_ and 0.5 mM for MgCl_2_. Reaction was started by adding of Pol ι to 25 nM final concentration and incubated at 37 °C for 10 min. The reaction was terminated by the addition of an equal volume of loading buffer (95% formamide, 10 mM EDTA, 0.1% bromophenol blue). DNA products were separated on 16% PAGE with 8 M urea.

## Electronic supplementary material

Supplementary Information
