# Supplementary material for: Identification of amino acid residues involved in the dRP-lyase activity of human Pol ι
Source: Sci Rep. 2017 Aug 31;7:10194. doi: 10.1038/s41598-017-10668-5 (PMC5579206; doi:10.1038/s41598-017-10668-5)
Supplement: Supplementary file 1 — Supplementary Information [file 41598_2017_10668_MOESM1_ESM.pdf]

Identification of amino acid residues involved in the dRP-lyase activity of human Pol ι

Nataliya Miropolskaya, Ivan Petushkov, Andrey Kulbachinskiy, Alena V. Makarova*

* corresponding author
Institute of Molecular Genetics, Russian Academy of Sciences, Kurchatov sq. 2, 123182 Moscow, Russia; tel: (499) 196 0015; E mail: amakarova-img@yandex.ru

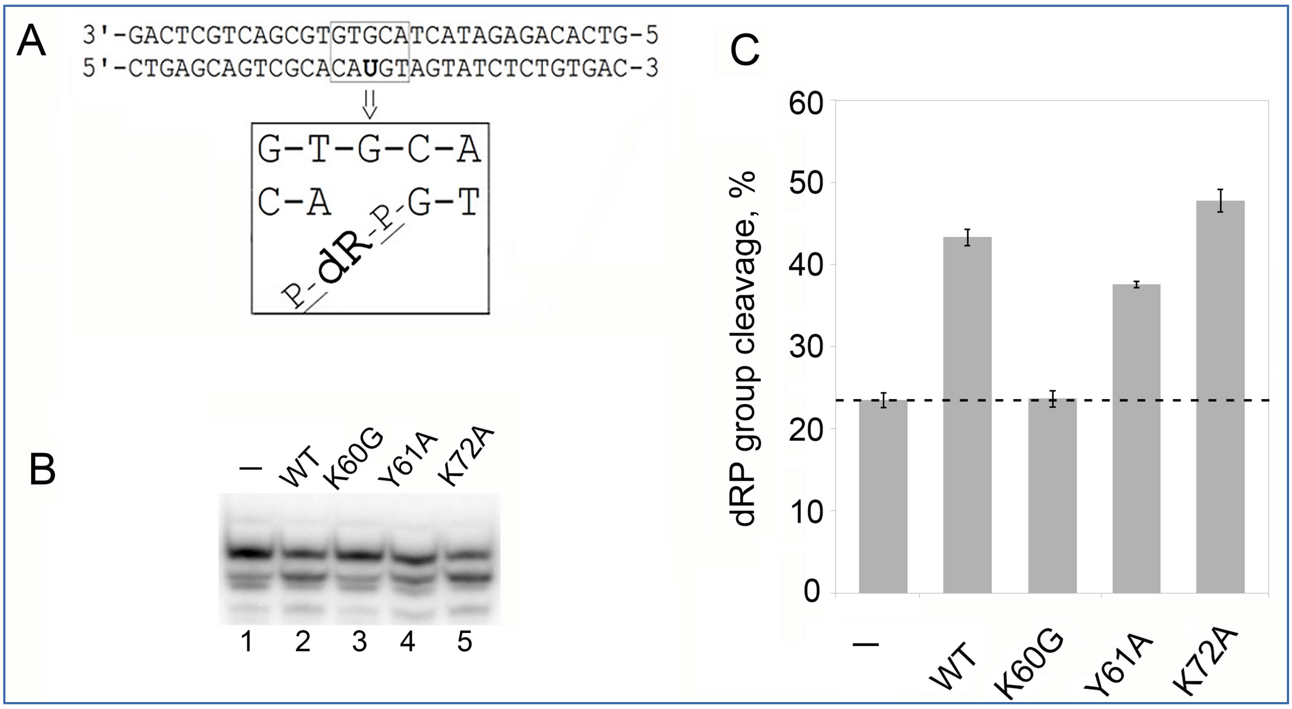

Supplementary Figure 1. Effects of Y61A and K72A amino acid substitutions on the dRP-lyase activity of Pol ι. (A) The structure of the oligonucleotide substrate with the 5'-dRP group used in this study. (B) Gel image of the dRP-lyase activity of wild-type and mutant Pol ι variants. Lane 1 ("-") show spontaneous cleavage of the 5'-dRP group observed in the absence of Pol ι protein. (C) Diagram showing percentages of the 5'-dRP group cleavage by Pol ι variants. The dashed line shows the level of spontaneous cleavage observed in the absence of Pol ι. Substitutions K60G, Y61A and K72A were obtained in the full-length Pol ι protein.

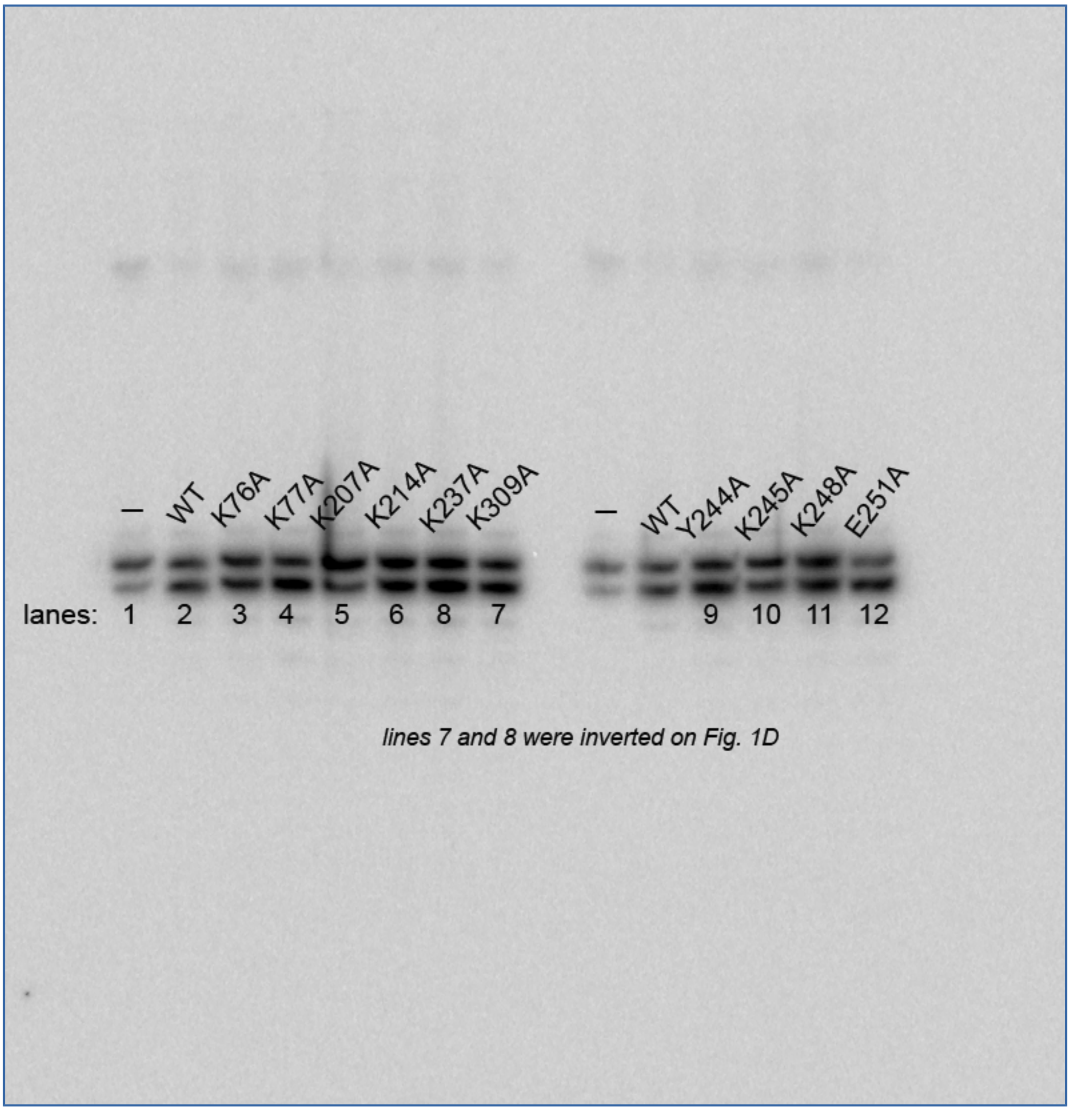

Supplementary Figure 2. The original gel image of Figure 1D, lanes 1 - 12

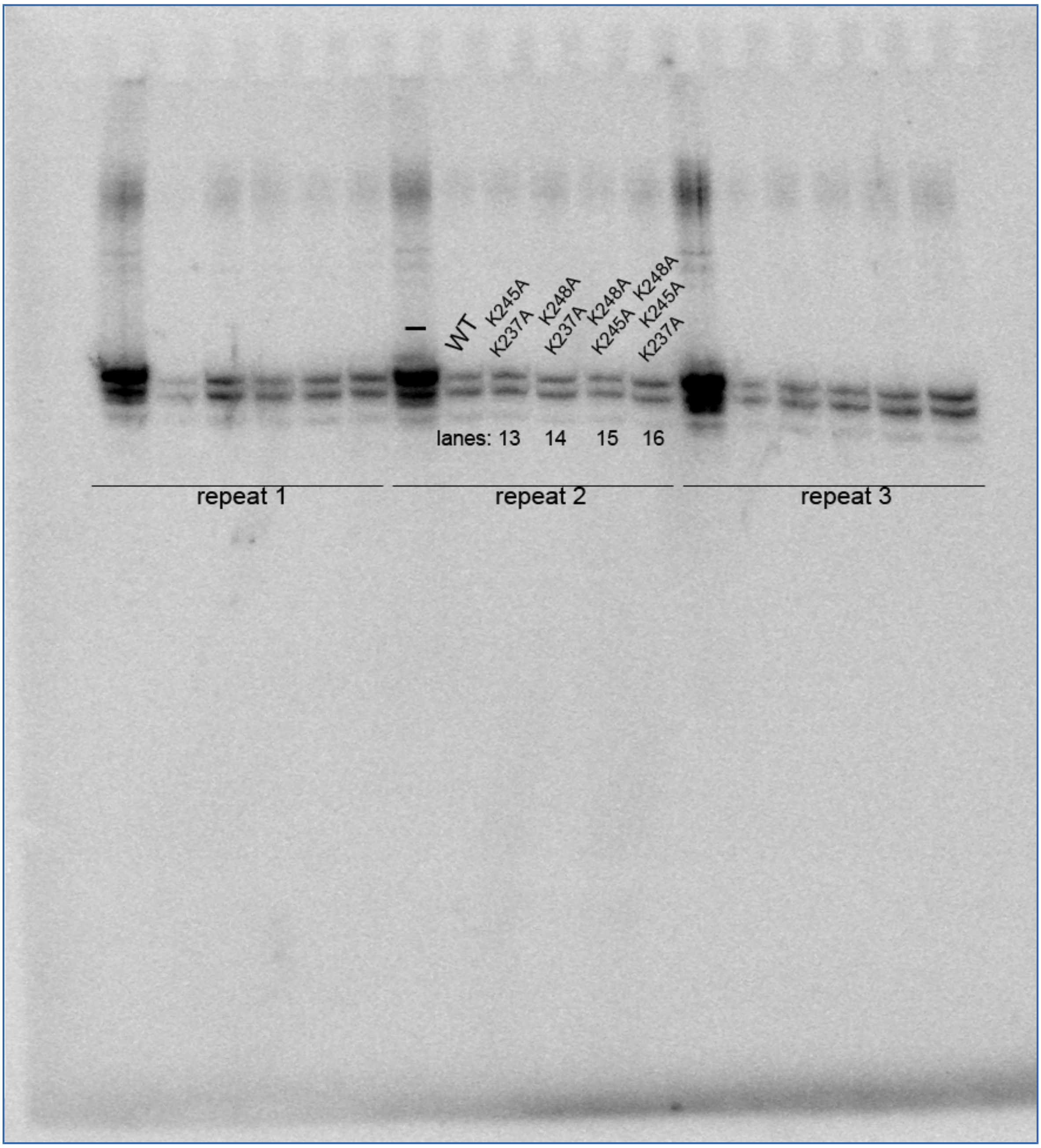

Supplementary Figure 3. The original gel image of Figure 1D, lanes 13 – 16.

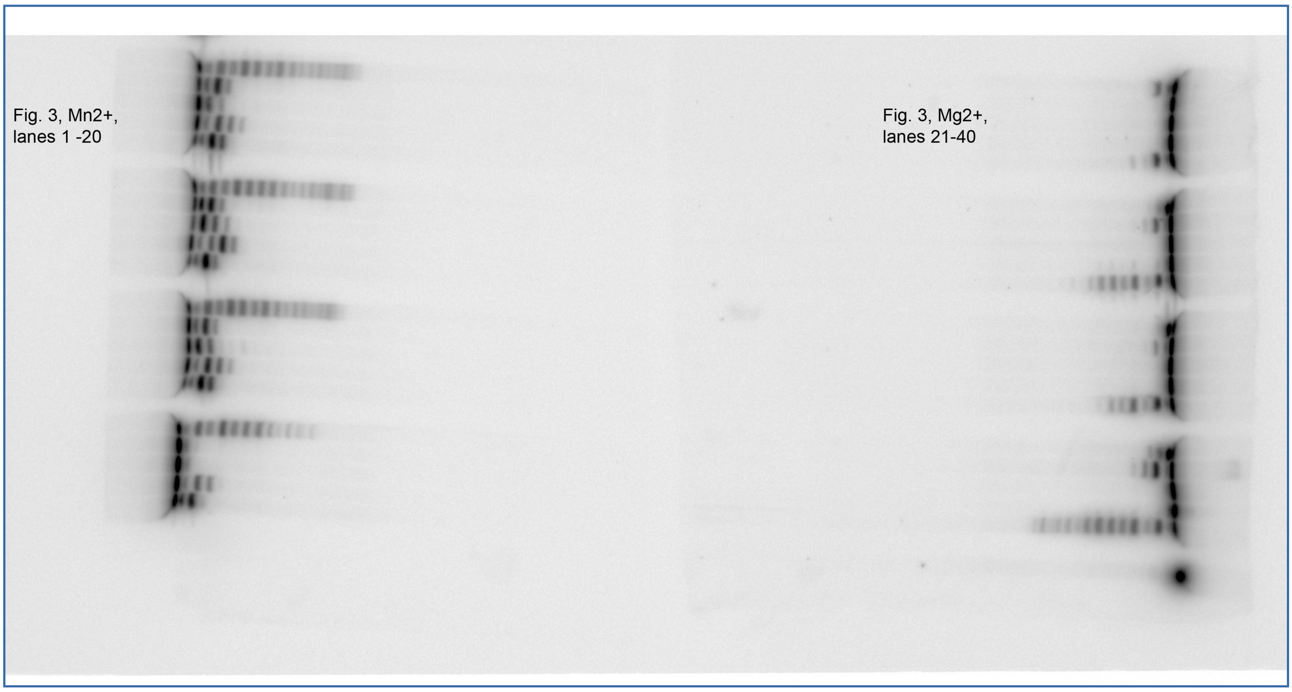

Supplementary Figure 4. The original gel image of Figure 3.
